# Silencing *Miniature* Gene Disrupts Elytral and Hindwing Structures in *Leptinotarsa decemlineata*

**DOI:** 10.3390/insects16070700

**Published:** 2025-07-08

**Authors:** Man-Hong Cheng, Kai-Yun Fu, Wei Zhou, Ji-Feng Shi, Wen-Chao Guo

**Affiliations:** 1 Institute of Plant Protection Xinjiang Academy of Agricultural Sciences/Key Laboratory of Integrated Pest Management on Crops in Northwestern Oasis, Ministry of Agriculture/Xinjiang Key Laboratory of Agricultural Biosafety, Urumqi 830091, China; chengmh0325@163.com (M.-H.C.); fukaiyun000@foxmail.com (K.-Y.F.); 2 College of Sericulture, Textile, and Biomass Sciences, Southwest University, Chongqing 400715, China; zorroice@yeah.net

**Keywords:** miniature, elytra, hindwing veins, *Leptinotarsa decemlineata*

## Abstract

The Colorado potato beetle (*Leptinotarsa decemlineata*, CPB) is a major pest in potato crops, notorious for its rapid dispersal and insecticide resistance, which are enabled by its robust elytra and flight-capable hindwings. The *Miniature* (*Mi*) gene, encoding a protein with a zona pellucida (ZP) domain, is involved in wing development and cuticle integrity, yet its functional role in beetles remains underexplored. In this study, we cloned and characterized the *LdMi* gene in the CPB and investigated its function using RNA interference (RNAi), morphological analyses, and spectroscopy. *LdMi* encodes a 146.35 kDa transmembrane protein with a conserved ZP domain, clusters with coleopteran homologs, and exhibits relative conservation across insect species. Expression profiling showed high *LdMi* transcript levels in the hindwings, the elytra, and the pupal stages. RNAi knockdown in fourth-instar larvae resulted in severe eclosion defects, including malformed wings and reduced adult weight. Scanning electron microscopy (SEM) revealed disrupted elytral patterns and deformed hindwing veins in knockdown individuals. Spectroscopic analyses using Fourier-transform infrared (FTIR) and Raman spectroscopy indicated a reduction in protein–chitin crosslinking and diminished hydrogen bonding, suggesting compromised cuticular integrity. These results highlight the essential role of *LdMi* in cuticle formation and the surface morphology of the elytra and hindwings, offering new insights into ZP domain proteins in insects.

## Simple Summary

The Colorado potato beetle (*Leptinotarsa decemlineata*) is a destructive pest that causes significant damage to potato crops. The beetle’s wings are crucial for its flight, and survival, and protection against environmental stressors, such as pesticides, which also make controlling this pest difficult. The *Miniature* (*Mi*) gene plays an essential role in the development of the wings and their protective outer covering (cuticle) in *Drosophila melanogaster*. In this study, we investigated the function of the *LdMi* gene in *L. decemlineata* using RNA interference to suppress its expression. Our results demonstrate that knockdown of the *LdMi* gene caused severe deformities in the elytra and hindwings of the beetle, with significant changes in the chemical composition of the wings, weakening their structural integrity. This study highlights the critical role of the *LdMi* gene in wing development and provides valuable insights into advancing pest control strategies.

## 1. Introduction

The Colorado potato beetle (*Leptinotarsa decemlineata*, CPB) is a notorious defoliator of potato crops and poses a serious threat to global potato production. Originally native to North America, the CPB has expanded its range across Europe, Asia, and Africa, causing significant crop losses [1,2,3,4]. In China, the beetle was first recorded in Xinjiang Province in the early 1990s and has since spread into Inner Mongolia, Heilongjiang, and Jilin, endangering additional potato-growing regions [5,6]. The beetle’s hindwings enable its strong flight capability, facilitating migration at speeds of 40–50 km per year, while the rigid elytra protect against environmental stress and act as a barrier to insecticides, reducing the effectiveness of chemical control methods [7,8,9,10,11]. This combination of flight mobility and elytral resistance presents considerable challenges for effective pest management.

The zona pellucida (ZP) domain is a conserved protein motif essential for insect development, particularly in remodeling the apical compartment, maintaining cuticle integrity, and facilitating wing morphogenesis [12,13]. ZP domain proteins are widely expressed in the epithelial tissues during development, significantly contributing to structural stability [12,13,14,15]. For example, in *Drosophila melanogaster*, ZP proteins like Dusky-like (Dyl) are essential for forming sensory bristles and wing hairs [16]. Additionally, the ZP proteins Papillote and Piopio (Pio) are involved in cell adhesion to the apical extracellular matrix and the organization of microtubules [17]. Research on other insects, such as *Henosepilachna vigintioctopunctata* and *Bombyx mori*, further emphasizes the diverse roles of ZP proteins in maintaining cuticle integrity and wing architecture [18,19]. In *Tribolium castaneum*, the ZP protein Dusky (Dy) functions upstream of Forked (Fork) in wing morphogenesis [20,21], while Dyl operates downstream of Blimp-1 through Shavenbaby (Svb) to regulate larval epidermal pigmentation and metamorphosis [22]. Moreover, knockdown of *TcSvb* significantly reduces the expression of related ZP domain proteins, such as Singed (Sg) [22].

The *Miniature* (*Mi*) gene, a member of the ZP domain protein family, is closely associated with cuticle strength and membrane resilience, both essential for insects’ flight performance and survival [12]. In *D. melanogaster*, the *DmMi* gene is critical to the differentiation and maturation of the wings’ epithelial cells. Studies have shown that *DmMi* and its homolog *Dusky* encode transmembrane proteins that localize to the apical membranes of wing epithelial cells, playing a significant role in cuticle formation and maintaining cellular integrity [23]. Furthermore, *DmMi* is associated with the neurohormone bursicon and forms a stable complex that facilitates wing cell maturation. Its expression is correlated with wing expansion and apoptosis in epithelial cells, with delayed wing expansion observed in *Drosophila* mutants lacking *Miniature* [24]. *DmMi* is also critical for post-eclosion wing maturation, influencing both apoptosis and the epithelial-to-mesenchymal transition (EMT) during wing development. The absence of *Miniature* compromises wing integrity and disrupts hormone diffusion within the extracellular matrix, leading to defects in wing morphology and function [25]. Despite these findings, the role of Mi in other insect species has yet to be reported.

In this study, we cloned and characterized the *LdMi* gene in *L. decemlineata* and assessed its expression across various developmental stages and tissues using quantitative real-time PCR (qRT-PCR). To investigate the function of *LdMi*, we employed RNA interference (RNAi), which revealed that the knockdown of *LdMi* significantly disrupted the morphology and structure of both the elytra and the hindwings. Additionally, we utilized scanning electron microscopy (SEM) and FTIR/Raman spectroscopy to evaluate the structural and compositional changes following the knockdown. Our findings provide valuable insights into the role of the *Mi* gene in beetle wing development and deepen our understanding of the functional diversity of ZP domain proteins across insect lineages.

## 2. Materials and Methods

### 2.1. Insect Collection and Rearing

*L. decemlineata* adults were collected from potato fields in Urumqi, Xinjiang (43.82° N, 87.61° E), during spring emergence and reared under the standard conditions [26]. The beetles were maintained at 28 ± 1 °C and 50–60% humidity under a 14:10 light–dark cycle, with fresh potato leaves provided as food. Eggs hatched within seven days, and the larvae passed through four instars: the first three lasted 2 days each, and the fourth lasted 3.5 days. The fourth-instar larvae then burrowed into the soil to pupate and emerged as adults.

### 2.2. Cloning of the LdMi Gene

The *LdMi* gene sequence was obtained from *L. decemlineata* genome (https://www.hgsc.bcm.edu/arthropods/colorado-potato-beetle-genome-project, accessed on 1 January 2024, PRJNA854273) and transcriptome databases (PRJNA464380). Total RNA was extracted using TRIzol reagent (Invitrogen, New York, NY, USA) according to the manufacturer’s instructions. RNA quantity and quality were assessed using a NanoDrop 2000 spectrophotometer (Thermo Fisher Scientific, New York, NY, USA), with purity determined by the OD260/280 and OD260/230 ratios. Gene verification was conducted via PCR using the primers listed in Table S1, followed by 5′- and 3′-RACE to obtain the full-length *LdMi* sequence. The RACE reactions were performed using the SMARTer RACE cDNA amplification kit (Takara Bio, Dalian, China). The full *LdMi* sequence was submitted to GenBank (Accession No. PQ374228).

The open reading frames (ORFs) and corresponding amino acid sequences were identified, with their theoretical isoelectric points, and the molecular weights were calculated using the ExPASy online tool (https://web.expasy.org/peptide_mass/, accessed on 1 January 2025). The exon–intron organization was predicted by aligning the ORFs with genomic sequences and visualized using Adobe Illustrator CS6. The domain analysis was performed using the SMART tool (http://smart.embl-heidelberg.de/ (accessed on 1 January 2025)). A phylogenetic tree was constructed using MEGA6 with neighbor-joining and 1000 bootstrap replicates. The sequence accession numbers for the ZP domain proteins from various insects are listed in Table S2.

### 2.3. Quantitative Real-Time PCR (qRT-PCR)

qRT-PCR was used to quantify the *LdMi* gene expression and evaluate the RNAi knockdown efficiency in *L. decemlineata*. Gene-specific primers (Table S1) were designed using Beacon Designer 7 (Premier Biosoft International, Palo Alto, CA, USA). cDNA was synthesized from various developmental stages and adult tissues to determine spatiotemporal expression profiles. For the analysis of the RNAi knockdown efficiency, the larvae were fed potato leaves treated with ds*LdMi* or ds*GFP* suspensions for three days, and samples were collected on day 3 to assess the knockdown levels compared to those in the controls.

Total RNA was extracted from the collected samples and reverse-transcribed into cDNA. qRT-PCR was conducted under the previously established conditions [26], using SYBR Green fluorescence for quantification. The gene expression levels were normalized to the geometric mean of the reference genes (RP18, RP4, ARF1, and ARF4), with the data analyzed via the 2^−ΔΔCT^ method. Each experiment was performed in triplicate.

### 2.4. dsRNA Preparation

Synthesis of the dsRNA targeting the *LdMi* transcripts was conducted utilizing the T7 dual promoter system, as per the established protocols [27]. Two non-overlapping regions of the *LdMi* gene were selected to ensure RNAi efficiency. Production occurred in the RNase III-deficient *E. coli strain HT115*, with induction using 0.1 mM IPTG. A dsRNA construct targeting enhanced green fluorescent protein (ds*GFP*) served as a negative control. Following induction, bacterial cultures were processed through centrifugation at 5000× *g* for 10 min, and the resulting pellets were resuspended in 0.05 M phosphate-buffered saline (PBS, pH = 7.4) to their original volume, with the final concentration of dsRNA at approximately 0.5 μg/mL for the bioassays.

### 2.5. dsRNA Delivery and RNAi Bioassays

The introduction of dsRNA into the *L. decemlineata* larvae followed the previously described methods [28]. Potato leaves were immersed in a dsRNA-containing bacterial suspension for five seconds, air-dried for two hours, and then used for larval feeding. The control groups were fed PBS- and ds*GFP*-treated leaves. Fourth-instar larvae, starved for four hours post-molting, were placed into feeding dishes. with ten larvae per dish and twelve replicates per treatment.

On day three, knockdown efficiency was evaluated through RT-qPCR in three replicates. The remaining larvae were fed untreated leaves to analyze the adult phenotypes. Once their prepupal behavior had been observed, the larvae were transferred into soil-filled cups for pupation. Seven days after pupation, the emerged adults were collected, and any eclosion defects, along with abnormalities in the elytra and the hindwings, were recorded and compared to the control groups.

### 2.6. Morphological Analyses of the Wings

Following *LdMi* knockdown, eclosion abnormalities, including deformities in the elytra and hindwings, were observed. No significant differences were found between the sexes, so females were selected for further analysis. Wing morphology was assessed by measuring the elytral and hindwing area, elytral areal density (defined as the elytron weight per area), and elytral thickness, following the established methods [28]. The elytra and hindwings from 4-day-old adults were imaged using a Leica DVM6 microscope (Wetzlar, Germany), and the area analysis was conducted using ImageJ software (Fiji version, Win64). The mid-elytral thickness was measured using a Mitutoyo micrometer, achieving a precision of ±0.001 mm. Although each elytron and hindwing was assessed independently, the statistical comparison revealed negligible differences between the left and right elytra, permitting pooling of the datasets for subsequent analyses.

### 2.7. SEM Analysis of the Elytra and Hindwings

The surface features of the dorsal and ventral sides of the elytra, the dorsal surface of the hindwings, and the hindwing veins in *L. decemlineata* following *LdMi* knockdown were examined using a Phenom scanning electron microscope (PHENOM SCIENTIFIC, Eindhoven, The Netherlands). To improve the conductivity, samples were coated with gold before imaging under high-vacuum conditions. Various regions of each elytron and hindwing were analyzed in detail to document the full range of structural abnormalities and fragility caused by *LdMi* suppression.

### 2.8. Spectroscopy Analysis

The chemical composition of both the elytra and the hindwings from female beetles with *LdMi* knockdown was analyzed using Fourier-transform infrared (FTIR) and confocal Raman spectrometry on micronized powder, obtained four days post-eclosion. Before spectral acquisition, lyophilized samples were ground into a fine powder and compressed into potassium bromide (KBr) pellets. The FTIR spectra were recorded using a Jasco 460Plus spectrometer (Jasco, Tokyo, Japan), operating at a resolution of 4 cm^−1^ over the range of 4000 to 400 cm^−1^, with 100 accumulations for each measurement. A Raman analysis was conducted using a WiTec Alpha 300R microscope (WiTec, Ulm, Germany), covering the spectral acquisition from 3500 to 100 cm^−1^ at a resolution of less than 0.5 cm^−1^. This system allowed for non-destructive biochemical profiling using 785 nm laser excitation, performing 100 scans of 500 ms each. All of the spectral data were processed for baseline correction, smoothing, and intensity normalization. Chitin derived from shrimp served as the reference standard for peak identification.

### 2.9. The Data Analysis

The data were analyzed using GraphPad Prism 10 software and presented as the mean ± SE. A one-way ANOVA was employed to assess the statistical significance across groups, followed by Tukey’s post hoc test for multiple comparisons. *p*-values were classified as *p* < 0.05, *p* < 0.01, and *p* < 0.001. Since no significant differences were detected between the two dsRNAs targeting different regions of the *LdMi* gene, the data were combined for the subsequent analysis.

## 3. Results

### 3.1. Characterization of the LdMi Gene in L. decemlineata

The *LdMi* gene fragments were identified through a comprehensive bioinformatics search across multiple transcriptome databases [29] and genomes [6,30] of *L. decemlineata*, utilizing the amino acid sequences of the *T. castaneum* and *D. melanogaster* Mi proteins as queries. Following molecular cloning and RACE techniques, we successfully obtained a full-length cDNA of 2195 nucleotides. The ORF comprises 1782 nucleotides, which putatively encodes a protein of 146.35 kDa and 593 amino acid residues.

**Figure 1:**
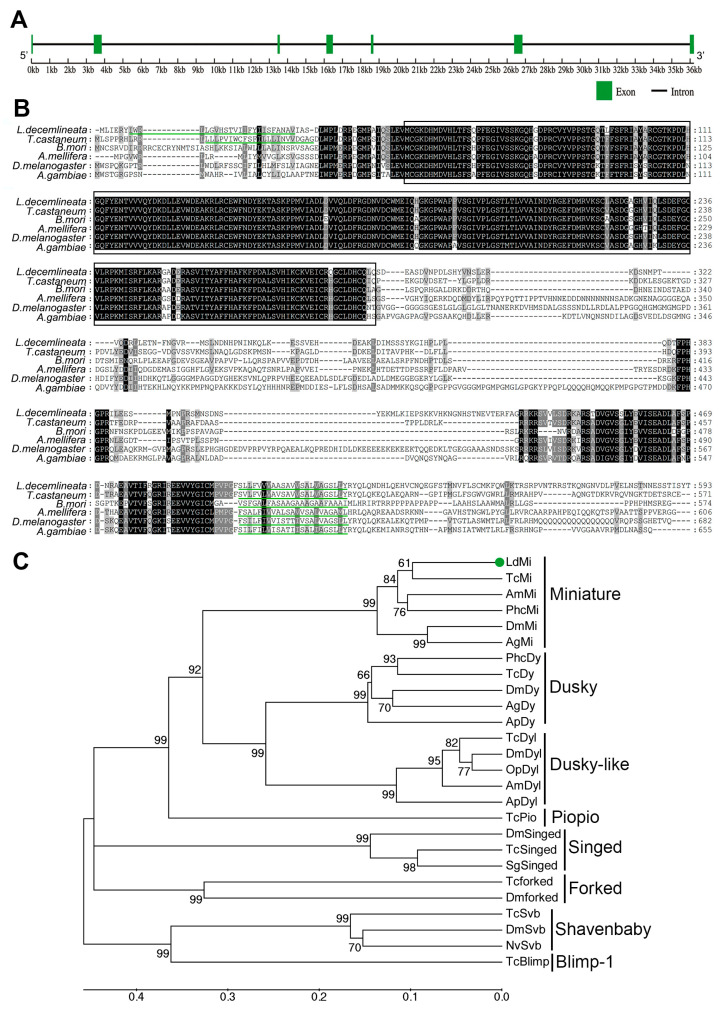
Gene structure, protein alignment, and phylogenetic analysis of the *Miniature* (Mi) protein. (**A**) A schematic of the exon–intron structure of the *LdMi* gene, where exons are shown as green-filled boxes and introns are indicated by lines. (**B**) Multiple sequence alignment of the Mi proteins highlights the ZP domain and the transmembrane region, delineated by black boxes and green lines, respectively. The shading intensity, from light to dark, reflects increasing sequence similarity. Alignment gaps were introduced to optimize the comparison. (**C**) A phylogenetic tree showing the evolutionary relationships of the Mi proteins and additional functionally characterized ZP domain proteins in insects, constructed using the neighbor-joining method with 1000 bootstrap replications. The tree includes eight groups: *Miniature* (Mi), Dusky (Dy), Dusky-like (Dyl), Piopio (Pio), Singed (Sg), Forked (Fork), Shavenbaby (Svb), and Blimp-1. The *L. decemlineata* miniature protein (*Ld*Mi) is indicated by a green dot.

Alignment of the full-length cDNA with the genomic DNA of *L. decemlineata* revealed the structure of the *LdMi* gene, consisting of seven exons (Figure 1A). Subsequent sequence alignment among the Mi proteins from *T. castaneum*, *B*. *mori*, *Apis mellifera*, *D. melanogaster*, and Anopheles gambiae indicated that Mi encodes a transmembrane protein characterized by a ZP domain (Figure 1B).

Phylogenetic analysis of the Mi proteins and other functionally characterized ZP-domain-containing proteins from various insect species, including Dy, Dyl, Pio, Sg, Fork, Svb, and Blimp-1, revealed distinct clustering patterns. The *Ld*Mi protein grouped closely with the other insect Mi proteins into a well-supported clade, suggesting a shared evolutionary origin and a conserved functional role across these species. Notably, *Ld*Mi clustered tightly with the *Tc*Mi protein from *T. castaneum* within the Coleoptera group. Additionally, the Mi proteins were more closely related to their paralogues, Dy and Dyl, than to the other ZP-domain-containing proteins. This implies that *Ld*Mi may share similar physiological functions with Dy and Dyl while potentially taking on new roles (Figure 1C).

### 3.2. The Expression of LdMi Across Developmental Stages and Tissues

**Figure 2:**
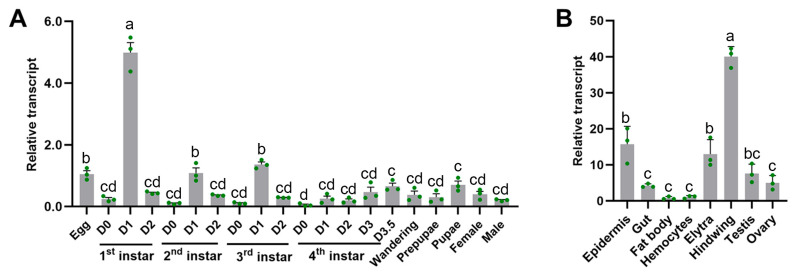
The transcription patterns of *LdMi* in *L. decemlineata*. (**A**) The temporal expression of *LdMi* was analyzed using cDNA templates prepared from pooled samples across various developmental stages, including mixed eggs (1–3 days old), first- to fourth-instar larvae (collected daily), wandering larvae, prepupae, pupae, and newly emerged adult males and females (day 1). The green circular dots indicate biological triplicates. (**B**) The tissue-specific expression was measured by quantifying the relative transcript levels in the epidermis, gut, fat body, hemocytes, elytra, and hindwings of day-1 adults, as well as the testes and ovaries of day-4 adults. Three independent pools of 20–30 individuals were analyzed for each sample, and qRT-PCR was employed, with technical triplicates. The 2^−ΔΔCt^ method was used to calculate the relative expression, normalizing against eggs (temporal analysis) or hemocytes (tissue analysis). The data represent the mean ± standard error (SE) of three biological replicates, with differing letters indicating significant differences (*p* < 0.05).

Before proceeding with the functional analysis of *LdMi*, we evaluated its mRNA levels across various developmental stages and tissues using quantitative real-time PCR (qRT-PCR) (Figure 2). During *L. decemlineata*’s developmental stages, *LdMi* was expressed in all of the samples analyzed, exhibiting a consistent expression pattern. Notably, the expression levels were lowest immediately after molting (day 0) and increased significantly during the mid-development stages of each instar (day 1) for the first to third instars and at the late stage of the fourth instar (day 3.5), with the first instar showing the highest expression on day 1 (Figure 2A). High expression levels were also observed during the pupal stage and at the egg stage. Tissue-specific analyses in adults revealed that the *LdMi* expression was highest in the hindwings, followed by the elytra and the epidermis (Figure 2B).

### 3.3. The Effects of LdMi Knockdown on Adult Eclosion and Wing Morphology in L. decemlineata

**Figure 3:**
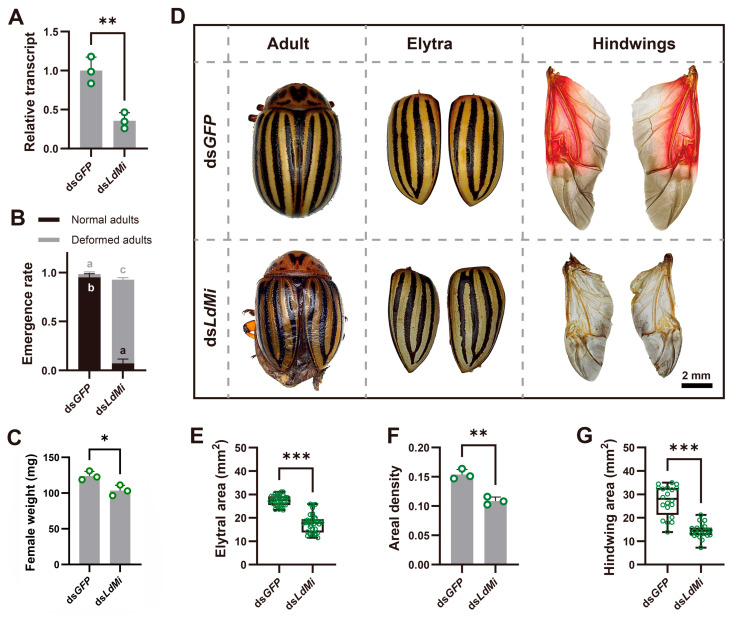
The impact of RNAi-mediated knockdown of *LdMi* on adult development and wing morphology in *L. decemlineata*. Fourth-instar larvae were fed potato leaves treated with ds*GFP* or ds*LdMi* for three days, followed by feeding with fresh leaves until pupation. (**A**) The transcript levels of *LdMi* were assessed on day 3 post-feeding, with green hollow circles representing biological replicates. Although *LdMi* knockdown did not affect pupation, it significantly increased the proportion of adults with deformities (**B**). Letters a, b, and c indicate the results from the one-way ANOVA, showing significant differences between the proportions of normally emerged and deformed females under the ds*LdMi* and ds*GFP* treatments. Female adults exhibited a reduced body weight on day 1 post-emergence (**C**). Knockdown individuals displayed morphological abnormalities, including irregularly shaped elytra and underdeveloped hindwings (**D**). The measurements of elytral area (**E**), areal density (**F**), and hindwing area (**G**) were significantly reduced. Asterisks denote statistically significant differences (* *p* < 0.05, ** *p* < 0.01, *** *p* < 0.001).

To investigate the function of *LdMi*, dsRNA specific to the *LdMi* gene was synthesized using *Escherichia coli HT115* and administered to newly molted fourth-instar larvae through the ingestion of treated potato leaves. The larvae were fed dsRNA-soaked leaves for three days, followed by fresh potato leaves until eclosion. ds*GFP* was used as the control. The qRT-PCR analysis indicated a significant reduction in *LdMi* mRNA levels following three days of continuous dsRNA intake (Figure 3A). The larvae treated with *LdMi* dsRNA showed a notable decrease in larval weight by day 4 (Figure S1). While normal pupation was observed (Figure S2), both male and female adults that emerged exhibited significantly lower weights compared to those in the ds*GFP*-treated group (Figure 3C and Figure S3). Furthermore, the eclosion rates declined significantly, primarily due to deformities in both the elytra and the hindwings (Figure 3B,D). Morphometric measurements indicated significant reductions in elytral area, areal density, and thickness (Figure 3D–F and Figure S4), as well as a marked decrease in hindwing area (Figure 3G).

### 3.4. Ultrastructural and Compositional Changes in the Elytra and Hindwings Following LdMi Knockdown

**Figure 4:**
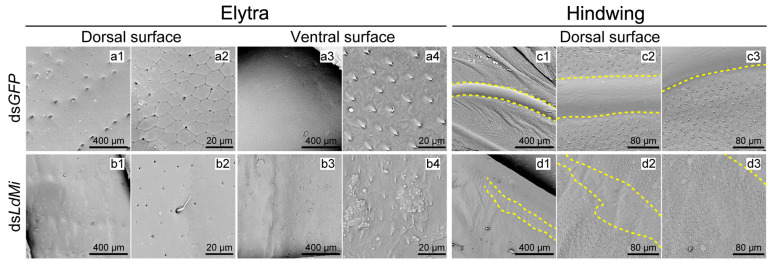
Ultrastructural defects in the elytra and hindwings of *L. decemlineata* following *LdMi* knockdown. In the ds*GFP*-treated beetles, the elytra displayed a regular hexagonal pattern on the dorsal surface (**a1**,**a2**) and a smooth, uniform ventral surface (**a3**,**a4**), with magnified views provided in panels (**a2**,**a4**). Hindwings exhibited a scale-like pattern with raised veins (**c1**–**c3**). In contrast, the ds*LdMi*-treated beetles showed a disrupted elytral structure, with a rough dorsal surface (**b1**,**b2**) and an irregular ventral surface (**b3**,**b4**). Knockdown individuals exhibited disorganized veins, flattened profiles, and blunted spiny structures in their hindwings (**d1**–**d3**). The boundaries of the hindwing veins are marked by yellow dashed lines.

The SEM analysis revealed clear morphological differences between the control (ds*GFP*) and *LdMi*-knockdown beetles (Figure 4). In the ds*GFP* group, the dorsal surface of the elytra exhibited smooth, well-defined polygonal patterns (Figure 4(a1,a2)), while the ventral surface contained uniformly arranged microstructures (Figure 4(a3,a4)). In contrast, *LdMi* knockdown resulted in irregular dorsal surfaces with collapsed regions (Figure 4(b1,b2)) and poorly organized ventral structures (Figure 4(b3,b4)). In the hindwings, ds*GFP*-treated individuals had smooth, raised veins and evenly distributed microtrichia across the membrane (Figure 4(c1–c3)). However, the *LdMi*-knockdown beetles exhibited flattened veins and sparse or missing microtrichia (Figure 4(d1–d3)), suggesting impaired wing development.

**Figure 5:**
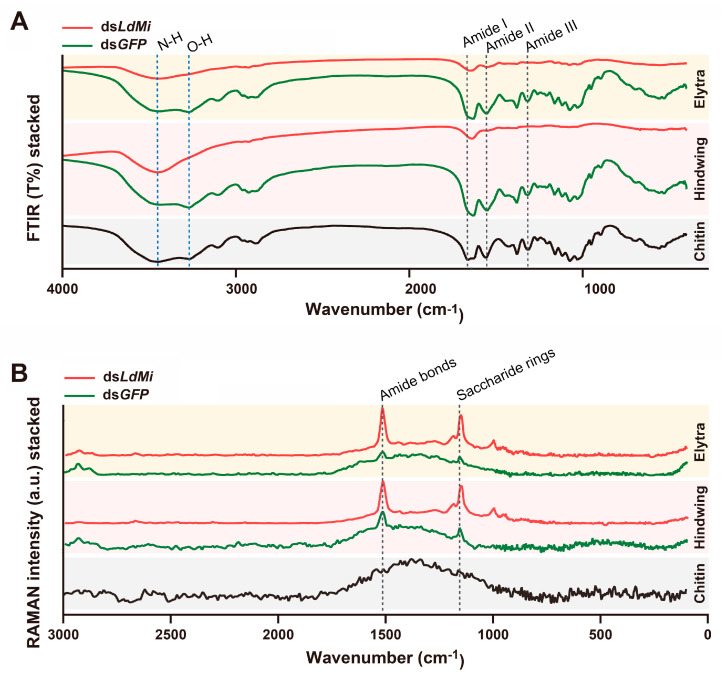
FTIR (**A**) and Raman (**B**) spectra analysis of compositional changes in *LdMi*-suppressed elytra and hindwings. (**A**) The gray dashed lines in the FTIR spectra highlight peaks for chitin and protein at 1650–1660 cm^−1^ (amide I), 1550–1560 cm^−1^ (amide II), and 1307 cm^−1^ (amide III). The black line indicates the reference standard for peak identification of chitin. Blue dotted lines indicate hydrogen bonding peaks at 3448 cm^−1^ (N-H stretching) and 3268 cm^−1^ (O-H stretching), linked to mechanical integrity. (**B**) In the Raman spectra, gray dashed lines mark peaks at 1158 cm^−1^ (saccharide rings) and 1515 cm^−1^ (amide bonds), reflecting chitin and protein structures.

To investigate the molecular and nanostructural changes associated with these morphological abnormalities further, FTIR and Raman spectroscopy analyses were performed on the elytra and hindwings of the *LdMi*-knockdown beetles (Figure 5). These spectra were compared to those for commercial chitin samples and the ds*GFP* control beetles to assess the impact of *LdMi* knockdown on the cuticular composition and structural integrity.

The FTIR absorption profiles of the ds*GFP* elytra and hindwings showed characteristic peaks for chitin (1550–1560 cm^−1^ for amide II, N-H bending and C-N stretching; 1650–1660 cm^−1^ for amide I, C=O stretching), proteins (notable peaks at 1550–1560 cm^−1^ and 1307 cm^−1^ for amide III), and lipids (2800–3000 cm^−1^) (Figure 5A) [31,32,33]. In contrast, the *LdMi*-knockdown samples displayed significant reductions in these peaks, especially in the amide II and amide III bands, as well as a marked decline in the O-H stretching peak at 3268 cm^−1^. These alterations indicate a disruption in hydrogen bonding and other structural interactions crucial for maintaining the mechanical integrity of the exoskeleton. Additionally, significant reductions across the fingerprint region (550–1300 cm^−1^) highlighted the degradation of chitin, proteins, and other structural components (Figure 5A) [34].

Raman spectroscopy further confirmed these findings. The control samples exhibited characteristic peaks at 1158 cm^−1^ and 1515 cm^−1^, corresponding to saccharide ring and amide bond vibrations, which are associated with the basic chitin and protein structures [33,35,36]. In contrast, the *LdMi*-knockdown samples showed intensified peaks at these positions (Figure 5B). This suggests abnormal overpacking and crystallization defects within the molecular structure, indicating significant changes in the organizational integrity of the cuticular components.

Together, these results highlight the crucial role of *LdMi* in maintaining the structural and molecular integrity of the elytra and the hindwings. *LdMi* knockdown leads to significant changes in both the ultrastructure and composition of these structures, impairing their mechanical properties and development.

## 4. Discussion

In this study, we cloned and characterized the *LdMi* gene, a member of the zona pellucida (ZP) domain protein family, in *L. decemlineata* [23,24,25]. RNAi-mediated silencing of *LdMi* resulted in wing abnormalities in adults, underscoring its critical role in wing morphogenesis. Below, we discuss the functional significance of *LdMi* in the context of wing development and cuticle formation.

### 4.1. Mi Is Conserved and Crucial for Wing Development

Multiple lines of evidence support the role of *LdMi* as a key regulator of wing development in *L. decemlineata*. The phylogenetic analysis revealed that *Ld*Mi belongs to a distinct clade of insect ZP domain proteins, closely related to Dy and Dyl (Figure 1C), which have previously been implicated in cuticle formation and epithelial morphogenesis [18,21,23]. This evolutionary relationship suggests conserved functional roles within this protein subfamily.

Tissue-specific expression profiling revealed that *LdMi* is predominantly expressed in the elytra and hindwing epidermis (Figure 2B), consistent with the findings in *D. melanogaster*, where *DmMi* orthologs are active in the developing pupal wings [23].

Moreover, RNAi-mediated knockdown of *LdMi* resulted in significant wing deformities, including reductions in their size and structural density (Figure 3). These findings are consistent with other studies in *Drosophila* species where Mi proteins regulated wing size and cellular structure [23,25]. For instance, mutations in *Mi* genes in *D. melanogaster* and *Drosophila virilis* lead to a 1.5-fold reduction in wing surface area [37,38]. In *DmMi* loss-of-function mutants, wing size is significantly reduced and the cuticle appears darker compared to these properties in the wild type [24]. Additionally, wing expansion and epithelial apoptosis are delayed, while the overexpression of Mi accelerates wing development [24], reinforcing the requirement for conserved Mi proteins for wing tissue organization.

Interestingly, despite its early-stage expression, *LdMi* knockdown did not visibly impact embryonic or larval epidermal development, suggesting that *LdMi* functions in a stage-specific manner, primarily during cuticle maturation and wing expansion. These observations are consistent with the findings in *Drosophila* and suggest that Mi may have additional roles, yet to be fully understood, during different developmental stages.

### 4.2. Mi Regulates Wing Surface Architecture and Cuticle Composition

In addition to gross morphological changes, silencing *LdMi* also disrupted the microstructure of the wing cuticle. SEM revealed collapsed and disorganized surface regions in both the elytra and the hindwings of the *LdMi*-knockdown beetles, including flattened veins and a marked reduction in microtrichia (Figure 4). These disruptions indicate a compromised apical extracellular matrix (ECM), which likely affects the integrity of the cuticulin envelope and its association with the cytoskeleton.

Similar morphological defects in wing structure and vein formation have been observed in *Drosophila* miniature (Mi) mutants. These mutants exhibit a reduced epidermal cell size, resulting in smaller wings, and impaired epidermal flattening during pupal development, preventing proper wing expansion [37,38]. This phenotype points to disrupted ECM organization, particularly within the cuticulin envelope, which is essential for wing shaping and mechanical support [23,39]. Disruption of Mi protein function compromises the coordination between the apical membrane, the cytoskeleton, and the ECM, resulting in defective cuticle formation and impaired wing expansion, as observed in both beetles and *Drosophila* [23,24,40].

Biochemical analyses using FTIR and Raman spectroscopy corroborated these structural findings further (Figure 5). Reductions in the amide I, II, and III peaks and attenuated signals for N–H and O–H stretching suggest a loss of protein–chitin crosslinking and hydrogen bonding—both critical for maintaining cuticle rigidity. Decreased intensity in the fingerprint region also indicates impaired chitin fiber organization, which may compromise the mechanical resilience of the wing cuticle.

These findings are consistent with previous reports on other insect models. For example, *Dy* and *Dyl*, which are structurally related to *LdMi*, have been shown to regulate chitin deposition and cuticular structure in *D. melanogaster* [16,23], *Harmonia vigintioctopunctata* [18], and *Tribolium castaneum* [20,41]. Knockdown of these genes leads to similar epidermal and cuticular defects, supporting the broader role of ZP domain proteins in cuticle biogenesis and epidermal differentiation.

## 5. Conclusions

This study demonstrate that *Ld*Mi is a crucial regulator of wing development and cuticular integrity in *L. decemlineata*. RNA-interference-mediated silencing of *LdMi* resulted in severe morphological deformities in both the elytra and the hindwings, leading to abnormal wing structures, a reduced adult body weight, and impaired eclosion. Spectroscopic analyses showed that these morphological defects were linked to compromised protein–chitin crosslinking and weakened hydrogen bonding, highlighting the gene’s role in maintaining both biochemical stability and mechanical strength.

## Data Availability

The original contributions presented in this study are included in the article/Supplementary Materials. Further inquiries can be directed to the corresponding authors.

## Supplementary Materials

The following supporting information can be downloaded at https://www.mdpi.com/article/10.3390/insects16070700/s1. Figure S1. Effect of *LdMi* gene knockdown on larval weight. Figure S2. Effect of *LdMi* knockdown on pupation rate. Figure S3. Effect of *LdMi* knockdown on male body weight. Figure S4. Effect of *LdMi* knockdown on elytral thickness. Table S1. Primers used in RT-PCR, RACE, ORF verification, dsRNA synthesis, and qRT-PCR. Table S2. GenBank accession numbers of representative ZP domain proteins.
