# Supplementary material for: Silencing Miniature Gene Disrupts Elytral and Hindwing Structures in Leptinotarsa decemlineata
Source: Insects. 2025 Jul 8;16(7):700. doi: 10.3390/insects16070700 (PMC12295875; doi:10.3390/insects16070700)
Supplement: Supplementary file 1 [file insects-16-00700-s001.zip › insects-3696625-supplementary.pdf]

Supplementary data

Silencing *Miniature* Gene Disrupts Elytral and Hindwing Structures in *Leptinotarsa decemlineata*

Man-Hong Cheng[1,†], Kai-Yun Fu[1,†], Wei Zhou[2], Ji-Feng Shi[2,*], Wen-Chao Guo[1,*]

1 Institute of Plant Protection Xinjiang Academy of Agricultural Sciences/Key Laboratory of Integrated Pest Management on Crops in Northwestern Oasis, Ministry of Agriculture/Xinjiang Key Laboratory of Agri-cul-tural Biosafety, Urumqi 830091, China; fukaiyun000@foxmail.com (K.-Y.F.)

2 College of Sericulture, Textile, and Biomass Sciences, Southwest University, Chongqing, 400715, China; chengmh0325@163.com (M.-H.C.); zorroice@yeah.net (W.Z.)

* Correspondence: shijifeng@swu.edu.cn; gwc1966@163.com (W.-C.G.)

† These authors contributed equally to this work.

Running Head: *Miniature* gene in *L.decemlineata*

Table of Contents:

Fig S1. Effect of *LdMi* gene knockdown on larval weight.

Fig S2. Effect of *LdMi* knockdown on pupation rate.

Fig S3. Effect of *LdMi* knockdown on male body weight.

Fig S4. Effect of *LdMi* knockdown on elytral thickness.

Table S1. Primers used in RT-PCR, RACE, ORF verification, dsRNA synthesis, and qRT-PCR.

Table S2. GenBank accession numbers of representative ZP domain proteins.

Supplementary

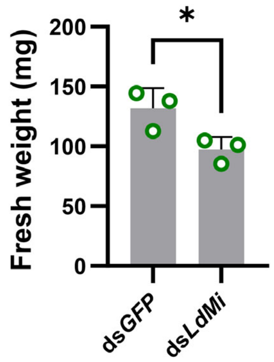

Figure S1 Effect of *LdMi* gene knockdown on larval weight. Fourth instar larvae were fed with dsLdMi for three days, resulting in a significant reduction in fresh weight on day 4 compared to the ds*GFP* control group. Bars represent mean values with error bars representing standard error (SE). Asterisks denote statistically significant differences ($p < 0.05$).

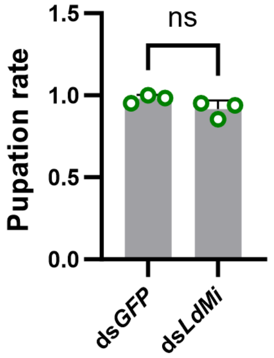

Figure S2 Effect of *LdMi* knockdown on pupation rate. No significant difference in pupation rate was observed between larvae treated with ds*LdMi* and those in the ds*GFP* control group.

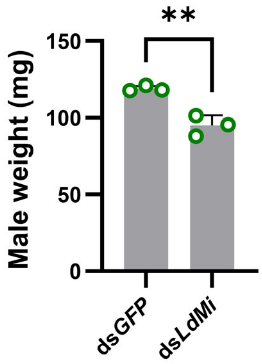

Figure S3 Effect of *LdMi* knockdown on male body weight. Male body weight was significantly reduced following *LdMi* knockdown compared to controls. Data are presented as mean ± SE, with ** indicating $p < 0.01$.

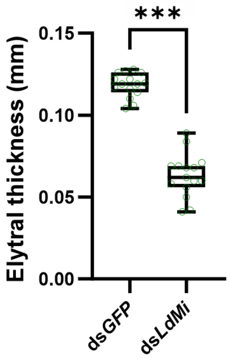

Figure S4 Effect of *LdMi* knockdown on elytral thickness. Knockdown of *LdMi* resulted in a significant reduction in elytral thickness compared to the ds*GFP* control group. Box plots display median, interquartile range, and individual data points. *** indicates $p <$ 0.001.

Supplementary Tables

Table S1 Primers used in RT-PCR, RACE, ORF verification, dsRNA synthesis, and qRT-PCR.

Fragment name	Forward primer	Reverse primer
**RT-PCR**		
LdMi-1	GCCAGCATTTCACGATTT	CACAGTGCCAGCCTCATA
LdMi-2	TCGCTAGTGATCTATGGC	GAAATGTATCCTGGAGGG
**RACE**		
LdMi 3'-GSP	AGGTGCGGACGAAAGAGC	
LdMi 3'-NGSP	AGAAAGATTCGCAGGCAGGAG	
**ORF verification**		
LdMi	ATATTTGGCGACTTCTAG	CGATTCGTTGTTAGTTGAA
**dsRNA synthesis**		
ds*LdMi-1*	ATGCCCAACCCCAGAAGTATG	TCCAACATCAGTGCTCCTTGC
ds*LdMi-2*	TTAGCCTCTGACGGAGCAGG	GCAGTGATCCAAACACCCTTGT
ds*GFP*	AAGTTCAGCGTGTCCG	CACCTTGATGCCGTTC
**qRT-PCR**		
q*LdMi*	GTCGTTTCCTCAAAGCACGA	GCAACTGGCAGTGATCCAAA
q*LdRP4*	AAAGAAACGAGCATTGCCCTTCCG	TTGTCGCTGACACTGTAGGGTTGA
q*LdRP18*	TAGAATCCTCAAAGCAGGTGGCGA	AGCTGGACCAAAGTGTTTCACTGC
q*LdARF1*	CGGTGCTGGTAAAACGACAA	TGACCTCCCAAATCCCAAAC
q*LdARF4*	GTGCTCGTGAACCATGTGAA	AACCTCCAATCCCTCGTGAA

Table S2 GenBank accession numbers of representative ZP domain proteins.

ZP domain Protein	Revised gene name	Accession number	Species
**Miniature**	*Tc*Mi	EFA00867	*Tribolium castaneum*
	*Bm*Mi	XP_004923535	*Bombyx mori*
	*Am*Mi	XP_392051	*Apis mellifera*
	*Dm*Mi	AAF48088	*Drosophila melanogaster*
	*Ag*Mi	EAA06905	*Anopheles gambiae*
**Dusky**	*Phc*Dy	EEB16849	*Pediculus humanus corporis*
	*Tc*Dy	EEZ99885	*Tribolium castaneum*
	*Dm*Dy	AAF48089	*Drosophila melanogaster*
	*Ag*Dy	EAA06947	*Anopheles gambiae*
	*Ap*Dy	XP_001952853	*Acyrthosiphon pisum*
**Dusky-like**	*Tc*Dyl	EFA12025	*Tribolium castaneum*
	*Dm*Dyl	AAF47884	*Drosophila melanogaster*
	*Op*Dyl	KOB66544	*Operophtera brumata*
	*Am*Dyl	XP_026298997	*Apis mellifera*

	*Ap*Dyl	XP_001944518	*Acyrthosiphon pisum*
**Piopio**	*Tc*Pio	URG87075	*Tribolium castaneum*
**Singed**	*Dm*Singed	CAA35585	*Drosophila melanogaster*
	*Tc*Singed	XP_972494	*Tribolium castaneum*
	*Sg*Singed	QVD39438	*Schistocerca gregaria*
**Forked**	*Tc*Forked	KYB25921	*Tribolium castaneum*
	*Dm*Forked	NP_001188660	*Drosophila melanogaster*
**Shavenbaby**	*Tc*Svb	AYV33765	*Tribolium castaneum*
	*Dm*Svb	CAD23206	*Drosophila melanogaster*
	*Nv*Svb	QBA17932	*Nasonia vitripennis*
**Blimp-1**	*Tc*Blimp	EFA04698	*Tribolium castaneum*
